# Quantitative Magnetic Resonance Imaging for Biological Image-Guided Adaptive Radiotherapy

**DOI:** 10.3389/fonc.2020.615643

**Published:** 2021-01-29

**Authors:** Petra J. van Houdt, Yingli Yang, Uulke A. van der Heide

**Affiliations:** ^1^ Department of Radiation Oncology, The Netherlands Cancer Institute, Amsterdam, Netherlands; ^2^ Department of Radiation Oncology, University of California, Los Angeles, CA, United States

**Keywords:** quantitative magnetic resonance imaging (MRI), biological image-guided adaptive radiotherapy, magnetic resonance imaging (MRI)-guided radiotherapy, functional magnetic resonance imaging (MRI), treatment response

## Abstract

MRI-guided radiotherapy systems have the potential to bring two important concepts in modern radiotherapy together: adaptive radiotherapy and biological targeting. Based on frequent anatomical and functional imaging, monitoring the changes that occur in volume, shape as well as biological characteristics, a treatment plan can be updated regularly to accommodate the observed treatment response. For this purpose, quantitative imaging biomarkers need to be identified that show changes early during treatment and predict treatment outcome. This review provides an overview of the current evidence on quantitative MRI measurements during radiotherapy and their potential as an imaging biomarker on MRI-guided radiotherapy systems.

## Introduction

At the turn of the century, two novel concepts were introduced in radiation oncology that acknowledged the complexity of tumor biology and that presented the challenges that must be met to improve the outcome of radiotherapy. Recognizing that tumors can respond rapidly to fractionated treatment, Yan et al. introduced the concept of adaptive radiation therapy (1). Instead of delivering the entire treatment with a single treatment plan based on pre-treatment imaging, the proposal was to create a closed-loop process where the treatment plan could be modified based on observed changes in the patient. To date, with state-of-the-art linear accelerators, on-board imaging equipment and software for image processing and treatment planning, we see this concept come to fruition (2, 3). The second concept, introduced by Ling et al., addressed the biological heterogeneity of a tumor (4). Using biological images that reveal metabolic, functional, physiological, genotypic, and phenotypic data, a biological target volume could be defined. This could be used to ‘paint’ a dose distribution that matched the biological heterogeneity. Since then, many imaging biomarker studies have been conducted, essentially trying to establish how radiosensitivity can be visualized non-invasively (5). It was shown that while tumors indeed are quite heterogeneous, this heterogeneity changes during the course of fractionated radiotherapy (6, 7).

At this stage, it becomes clear that, considering the biological characteristics of the tumor as well as its dynamic nature during treatment, the two concepts of biological targeting and adaptive radiotherapy need to be merged. Based on frequent imaging, monitoring the changes that occur in volume, shape as well as biological characteristics, a treatment plan can be updated regularly to accommodate the observed response (8). While the logistical challenges for biological image-guided adaptive radiotherapy (BIGART) made the concept almost infeasible to carry out in practice, the emergence of MRI-guided radiotherapy (MRIgRT) platforms may be a game changer (9, 10).

For this purpose, imaging biomarkers need to be identified that show changes early during treatment and predict treatment outcome. Quantitative MRI (qMRI) techniques can be used to assess tumor morphology, biology and function. Therefore, they are promising imaging biomarkers for BIGART (9). In this review, we summarize the current evidence on repeated qMRI measurements during radiotherapy and the potential for such an approach with MRIgRT systems.

## Quantitative Manetic Resonance Imaging Biomarkers

The majority of MRI biomarker studies investigate the potential of a measurement prior to the onset of treatment to predict outcome (11–13). In addition, promising evidence has emerged showing changes in qMRI values during radiotherapy. This suggests that qMRI parameters are prognostic for outcome and might be potential biomarkers for BIGART (9). In this section the literature is discussed in which measurements during the course of radiotherapy were reported (**Table 1**). Studies with only pre- and post-treatment measurements were out of the scope of this review.

**Table 1:** Summary of MR imaging techniques for which changes during the course of radiotherapy have been investigated.

MR imaging technique	qMRI metric	Tissue characteristics	Studies investigating changes during RT
**DWI**	ADC	Tissue cell density	Rectum (14–25) Cervix (26–39) Head-and-neck (40–48) Esophagus (49–57) Brain (58) Lung (59) Liver (60) Prostate (61, 62) Sarcoma (48)
**DCE-MRI**	semi-quantitative measurements (e.g. peak enhancement) quantitative parameters derived with pharmacokinetic modeling (e.g. K^trans^, v_e_)	Perfusion and vascular permeability of tumor microenvironment	Cervix (32, 63–65) Head-and-neck (44, 66, 67) Esophagus (50) Liver (68)
**IVIM**	f, D, D*	Tissue perfusion and cell density	Cervix (69–74) Esophagus (75, 76) Head-and-neck (77, 78) Brain (79, 80)
**Relaxometry**	T2, T1, PD, T2*	Tissue relaxation times	Prostate (61, 62) Brain (81, 82)
**Spectroscopy**	e.g. choline to creatine ratio	Metabolism	Brain (83–86) Cervix (35) Head-and-neck (87)
**OE-MRI**	O_2_ concentration	Hypoxia	Lung (88)
**Saturation transfer MRI (MT, CEST)**	e.g. MTR, qMT, MTR_asym,_ MTR_amide_	Tissue macromolecular content (e.g. lipids, proteins, peptides)	Brain (81, 89, 90) Head-and-neck (91)
**Fat composition**	PDFF, %PDFF	Fat content	Bone marrow (92)
**Radiomics**	Histogram features, local textural features	Tissue heterogeneity	Cervix (93, 94) Rectum (95, 96) Head-and-neck (97) Sarcoma (98) Pancreas (99)

Papers were searched on PubMed with search terms “early response” and “radiotherapy” or “radiation oncology” as well as measurements during treatment mentioned in title or abstract. Only studies in humans and in English were included. Reference list of the included papers were checked to identify other relevant papers.

DWI, diffusion-weighted imaging; ADC, apparent diffusion coefficient; DCE-MRI, dynamic contrast-enhanced MRI; K^trans^, volume transfer constant between blood plasma and extravascular extracellular space; v_e_, fractional volume of extravascular extracellular space; IVIM, intravoxel incoherent motion imaging; f, perfusion fraction; D, diffusion coefficient; D*, pseudo-diffusion coefficient; T2, T2 relaxation time mapping; T1, T1 relaxation time mapping; PD, proton density mapping; R2*, R2* mapping; OE-MRI, oxygen-enhanced MRI; MT, magnetization transfer; CEST, chemical exchange saturation transfer; MTR, magnetization transfer ratio; qMT, quantitative magnetization transfer; MTR_asym_, magnetization transfer asymmetry; MTR_amide_, magnetization transfer ratio of amide protons; PDFF, proton density fat fraction.

Diffusion weighted imaging (DWI) has been the most investigated technique so far. The apparent diffusion coefficient (ADC) derived from DWI data has been associated with the cell density of the tissue. Radiotherapy results in breakdown of cellular membranes and finally necrosis (13, 100). As a result the cell density is reduced, which will be observed as an increase in ADC. For many tumor sites, changes in ADC parameters early during radiotherapy have been reported, including rectal cancer (14–20), cervical cancer (26–38), head and neck cancer (40–47), esophageal cancer (49–56), brain cancer (58), lung cancer (59), and liver cancer (60). The majority of the studies report a larger increase in average ADC values for responders compared to non-responders (15–20, 29, 33, 36, 41, 44–47, 49, 51, 54–56, 60). Some studies observed a significant increase for responders and not for non-responders (14, 35, 43). Only a few studies did not observe a significant difference in the changes in ADC values between responders and non-responders (34, 52). For example, in a study with 108 cervical cancer patients there was no difference in the increase in ADC values between complete and partial responders (34).

Dynamic contrast-enhanced (DCE-) MRI indirectly measures the tissue perfusion and vascular permeability of the tumor microenvironment and has been proposed as a biomarker for radiotherapy (101, 102). The enhancement reflects the abnormal microvasculature in tumors (102). Changes during treatment in DCE-MRI have been investigated to a lesser extent than DWI. Most studies have been performed for cervical cancer (32, 63–65). One of the first studies showed with a semi-quantitative analysis that an increase in enhancement early during treatment was predictive for local recurrence (63). Gong et al. observed similar results, as they found a significant relation between the change in mean enhancement and tumor regression rate (64). This was confirmed in a larger patient population showing that patients with an improved perfusion during treatment have a more favorable outcome (65). Quantitative analysis of DCE-MRI data showed an increase in K^trans^ (volume transfer constant between blood plasma and extravascular extracellular space) and v_e_ (fractional volume of extravascular extracellular space) during treatment, both in week 1 and week 4 (32). K^trans^ decreased 1 month after treatment again. The changes in K^trans^ and v_e_ during treatment were not correlated to changes in tumor volume. In a small group of head and neck cancer patients a larger increase in K^trans^ and v_e_ was observed in responders than in non-responders (44). Similarly Baer et al. reported that changes in K^trans^ and the area under the curve were predictive for survival (66). In addition, patients that have large persistent subvolumes with low blood volume within the primary tumor have a higher probability of local failure (67). For esophageal cancer, a decrease in K^trans^ was reported in complete responders (50). For liver metastases, an increase in slope and peak at week 2 was associated with an improved local response (68).

A limitation of DCE is that contrast agent needs to be injected intravenously. This could present logistical challenges and might not be amenable for repeated imaging. Alternatively, intravoxel incoherent motion (IVIM), based on multi-b-value diffusion, has been investigated for probing microscopic perfusion (103). By modeling the diffusion data with a perfusion component that predominantly affects low b-value data, a surrogate for tissue perfusion can be calculated (104). Studies in cervical cancer have reported changes in IVIM parameters during treatment (69–74). The perfusion fraction (f) first increased early during treatment and decreased later during treatment (72). Early increases in f have been associated with good response (70, 73). In esophageal cancers, responders showed a larger mid-treatment increase in the diffusion coefficient (D) of the tumor compared to non-responders (75, 76). Head-and-neck cancer patients with regional failure showed higher D values and larger reductions in f than patients with regional control (77).

For other qMRI techniques changes during treatment have been investigated only on a small scale so far. Spectroscopy has mainly been applied in brain (83–86). Changes in choline and lactate metrics during treatment were significantly related to outcome in patients with glioblastoma (83) and glioma (84). In two other studies only changes after treatment were significantly related to outcome (85, 86). For cervical cancer, changes in choline metrics could not predict treatment outcome (35). For head-and-neck cancer, choline metrics were stable in the first two weeks of treatment in responders and non-responders (87). Magnetization transfer (MT) and chemical exchange saturation transfer imaging (CEST) can be used to characterize the macromolecular content of tissue (105, 106). Changes during treatment have been investigated in glioblastoma (81, 89, 90) and head-and-neck cancer (91). All studies demonstrate the promising value of MT or CEST parameters as possible biomarkers for BIGART. Another promising technique is oxygen-enhanced MRI requiring an oxygen challenge (107). This technique was used in lung cancer patients to assess the hypoxic volume in the tumor (88). In the second week of the treatment the hypoxic volume was smaller than before treatment. Fat quantification could be useful to assess changes in tissue composition. For example, changes in fat fraction were correlated with changes in bone marrow composition induced by radiotherapy (92), which could be useful to assess hematologic toxicity. A few studies have looked into the potential of radiomics, where textural features derived from anatomical or functional images were tested (93–95, 97). Recently, deep learning approaches have been applied to extract information from images during treatment for response prediction (95, 108).

The evidence so far is mostly based on one or two measurements during treatment. Only a few studies used more than two measurements during treatment (**Table 2**). The study of Sun et al. showed in a population with mixed tumor sites that changes in ADC were correlated with treatment response and independent of tumor location (21). After the first week of treatment significant differences between responders and non-responders were observed, while a change in tumor size was not visible that early. In a study with cervical cancer patients, measurement of ADC at two weeks seemed optimal for monitoring early treatment response (39). Similar results were found for esophageal cancer (57) and rectal cancer (22). In contrast, a study with nine rectal cancer patients reported a decrease in ADC from week 2 onwards (23). Two studies investigated weekly changes in T2 and ADC values during treatment of prostate cancer (61, 62). While there were differences in overall treatment duration between the two studies, both studies did not observe early changes in either T2 or ADC. Only late ADC changes for the tumor were observed. However, the relation with treatment outcome was not assessed. A study in head-and-neck cancer patients investigated whether changes in IVIM parameters were visible during treatment (78). They showed a significant increase in ADC and D during treatment for patients with complete response. No significant differences were observed for the other IVIM parameters in the complete responding or non-responding patients.

**Table 2:** Overview of studies with more than two measurements during treatment.

Paper	MRI Technique	Tumor site	No. patients	No. time points
**Diagnostic scanners**				
Sun et al. (21)	DWI	Lung, esophagus, gastric, rectum, and liver metastases	102	Pre, w1, w3, w6 or pre, w1, w2, w4
Liu et al. (39)	DWI	Cervix	33	Pre, d3, d7, d14, 1m, and post
Wang et al. (57)	DWI	Esophagus	38	Pre, weekly (6x)
Cai et al. (22)	DWI	Rectum	15	Pre, weekly (5x)
Hein et al. (23)	DWI	Rectum	9	Pre, weekly (4x)
Foltz et al. (61)	DWI, T2	Prostate	17	Pre, w2, w4, w6, w8
Van Schie et al. (62)	DWI, T2	Prostate	47	Pre, every fraction (5x)
Paudyal et al. (78)	IVIM	Head-and-neck	34	Pre, weekly (3x)
Mahmood et al. (79)	IVIM	Brain metastases	29	Pre, every fraction (10x), post
Mahmood et al. (80)	DWI	Brain metastases	21	Pre, every fraction (10x), post
Bostel et al. (24)	DWI	Rectum	8	Every fraction (28x)
**MRIgRT systems**				
Yang et al. (48)	DWI	Head-and-neck, sarcoma	6	Pre, every 2-5 fractions (4-7x)
Shaverdian et al. (25)	DWI	Rectum	3	Every 3-7 fractions (4-7x)
Nejad-Davarani et al. (82)	T1, PD, R2*	Brain metastases	4	Pre, weekly (7x), post
Gao et al. (98)	DWI	Sarcoma	30	Fraction 1, 3, and 5
Boldrini et al. (96)	T2*/T1*-weighted	Rectum	16	Pre, weekly (5x)
Simpson et al. (99)	T2*/T1-weighted	Pancreas	20	Every fraction (5x)

Papers were searched on PubMed with search terms “early response” and “radiotherapy” or “radiation oncology” as well as measurements during treatment mentioned in title or abstract. Only studies in humans and in English were included. Reference list of the included papers were checked to identify other relevant papers. From those only papers with more than two measurements during treatment are presented in this table. Pre, pre-treatment; d, day; w, week; m, month; post, post-treatment; DWI, diffusion-weighted imaging; IVIM, intravoxel incoherent motion imaging; T2, T2 relaxation time mapping; T1, T1 relaxation time mapping; PD, proton density mapping; R2*, R2* mapping.

Up to this moment, only three studies performed daily measurements during treatment in humans (24, 79, 80). Mahmood et al. performed daily IVIM measurements in patients with brain metastases. They showed that the mean ADC increased for patients with responding brain metastases and decreased for non-responding metastases (79). From fraction seven onwards the distinction between responders and non-responders became more pronounced. The IVIM parameters, perfusion fraction f and pseudo-diffusion coefficient D^*^, did not show significant prognostic value. In another study, they showed that the size of the viable tumor delineated on DWI images and the ADC value of the viable tumor are a better predictor for outcome than the change in tumor size delineated on anatomical images (80). In a small, but unique, study with 8 rectal cancer patients, ADC values during treatment overlapped between complete and partial responders (24). Therefore, no significant differences in ADC dynamics were observed between the two groups.

The small number of studies with multiple measurements per patient may be explained by logistical challenges and the cost of MRI exams beyond standard-of-care. Here, the MRIgRT systems provide an opportunity. For patients who are treated on an MRIgRT system the logistical barrier is much lower as it only requires some prolonged time for imaging on the table (9). In fact, as the online adaptive workflow on MRIgRT systems takes up some time, quantitative imaging can be acquired during this time period, avoiding an increase in overall time on the table. As MRIgRT systems have been introduced in clinical practice recently (10), only a few qMRI studies have been performed so far. Feasibility of qMRI on MRIgRT systems was first demonstrated in a pilot DWI study. In this study, longitudinal DWI was acquired from a cohort of patients with head-and-neck cancer and sarcoma every 2-5 fractions throughout their treatment courses with different ADC change patterns observed (48). In a similar way, the feasibility of DWI for response assessment was shown in three rectal cancer patients (25). A pilot with four patients with brain tumors showed that changes in T1, R2* and proton density maps were detectable during the course of treatment (82). In addition, a few studies assessed the feasibility of using radiomic features to monitor response during treatment (96, 98, 99). For sarcoma patients it was shown that radiomic features derived from longitudinal DWI can be used to predict post-surgery tumor necrosis score after radiotherapy (98). The study of Boldrini et al. illustrated that changes in radiomic features during treatment have the potential to predict clinical complete response in rectal cancer (96). In addition, a pilot study showed that radiomic features could predict outcome for patients with pancreatic cancer treated with stereotactic ablative body radiotherapy on an MRIgRT system (99).

## Technical Validation

To integrate an MRI and a linear accelerator, modifications have been made to the MRI scanners in these systems. As a result, their technical specifications differ considerably from those of diagnostic systems. For the MRIdian (Viewray Technologies Inc. USA), the on-board MRI is a split bore superconducting magnet with a field strength of 0.35 T (109, 110). There is a 28 cm gap in between to reduce the number of MR components being in the radiation beam pathway. In case of the Unity system (Elekta AB, Sweden), the field strength is 1.5 T, but the gradient coils are physically split to create a radiation window (111). The 2 x 4 channel receive coil is radiolucent with all electronic components at the edges of the coil (111). The reduced signal-to-noise ratio and gradient performance for both systems put constraints on the acquisition protocols and the performance of qMRI measurements. Therefore, first efforts have been taken to assess the performance of these measurements on MRIgRT systems with phantoms (48, 82, 112–114). For the MRIdian 0.35T MRI, a few DWI studies have been performed, demonstrating the ADC accuracy and reproducibility, as well as improving DWI spatial integrity (48, 112). Studies of Nejad-Davarini et al. (82) and Bydder et al. (113) also explored feasibility and accuracy of T1 mapping, R2* mapping, proton density mapping, and proton density fat fraction using MRIdian. A multicenter study showed that consistent ADC, T1, T2, and DCE values can be measured across institutes with a Unity system (114). The accuracy of the techniques was similar to previously reported literature on diagnostic scanners. In addition, the feasibility of these qMRI techniques was demonstrated for a prostate cancer patient. Phantom measurements showed that accurate ADC values can be obtained within a 7 cm radius of the iso-center (115). Outside this region, ADC values deviated more than 5%. To increase the time window during which qMRI data can be acquired, the effect of image acquisition during irradiation has also been investigated. Phantom images acquired during gantry rotation were negligibly different from images with a static gantry (116). However, bulk shifts in the order of one pixel were observed and the extent of the phantom was gantry angle dependent. Therefore, DWI with an echo planar imaging sequence may require special attention to geometrical shifts and distortions. With test-retest measurements in prostate cancer patients it was shown that the rotating gantry did not affect the repeatability of ADC measurements (115).

## Discussion

With BIGART two important concepts in radiotherapy are brought together. Recognizing the dynamic heterogeneity of a tumor during radiotherapy and adapting the treatment to the changing characteristics may widen the therapeutic window between tumor control and treatment-related toxicity. Although the two concepts have been around for over two decades, only now the technology is available to integrate daily biological imaging with online treatment adaptation. While many qMRI biomarker studies have been conducted, many more steps need to be taken before BIGART on MRIgRT systems becomes routine practice.

From a clinical perspective, the first step will be to investigate daily changes in qMRI values in different tumor sites. Multicenter observational trials should be initiated to validate these findings. In particular, it is important to investigate which qMRI techniques are suitable candidates for BIGART (117, 118). Based on the current and mostly consistent evidence, DWI seems to be a logical first choice to investigate further. The potential of DCE needs to be established, but might be very useful in certain applications (102). IVIM is an attractive alternative to study perfusion as it avoids administration of a contrast agent. Although previous studies observed a weak to moderate correlation between DCE and IVIM parameters (119–123), for BIGART it might be sufficient if similar trends are visible in the IVIM and DCE parameters. Other qMRI techniques are also promising, but must be investigated with larger populations. As different qMRI techniques reflect different aspects of tumor biology, a combination of techniques might give complimentary information with a higher predictive value for early treatment response (50, 53). Another open issue is the time scale at which changes in qMRI values happen during treatment. Some studies have reported changes early during treatment, others later. Monitoring changes on a daily basis, will help characterize this further. In addition, this will also reveal whether changes are homogeneous at group level (e.g. responder or non-responder groups), whether the time scale of the changes differs on patient-level or even differs within the tumor of the same patient. Furthermore, the relevance of observed changes in relation to treatment outcome (e.g. survival, recurrence, toxicity) needs to be established in order to identify if a biomarker potentially is predictive and suitable for BIGART.

Technical validation (124–126) of qMRI measurements on MRIgRT systems is required to ensure that the results are also relevant outside the MRIgRT domain, in particular because the MR-part of the MRIgRT systems is different from diagnostic systems. Digital and physical phantoms can be used to assess the accuracy and reproducibility of the qMRI measurements (127–134). Furthermore, to know which changes in qMRI values can be attributed to the effect of the treatment, assessment of the repeatability of the measurements should be performed with test-retest studies (125). Standardization of qMRI protocols could assist to improve reproducibility across participating centers (115).

In conclusion, MRIgRT systems have the potential to bring adaptive radiotherapy and biological targeting together in practice. The first step will be to investigate daily changes in qMRI values in different tumor sites, validated in a multicenter setting. Then, interventional studies become feasible to investigate the potential of qMRI as a biomarker for BIGART.

## Author Contributions

PH and UH contributed to the conception and design of the review paper. All authors contributed to the article and approved the submitted version.

## Conflict of Interest

The Netherlands Cancer Institute is a member of the Elekta Unity consortium and receives research funding from Elekta AB (Sweden) and Philips Healthcare (the Netherlands).

The authors declare that the research was conducted in the absence of any commercial or financial relationships that could be construed as a potential conflict of interest.
